# Insights into Surface Interactions between Metal Organic Frameworks and Gases during Transient Adsorption and Diffusion by In-Situ Small Angle X-ray Scattering

**DOI:** 10.3390/membranes6030041

**Published:** 2016-09-03

**Authors:** Ludovic F. Dumée, Li He, Peter Hodgson, Lingxue Kong

**Affiliations:** Institute for Frontier Materials, Deakin University, Geelong 3216, Australia; li.he@deakin.edu.au (L.H.); peter.hodgson@deakin.edu.au (P.H.); lingxue.kong@deakin.edu.au (L.K.)

**Keywords:** in-situ SAXS, metal organic frameworks membranes, carbon nanotube bucky-papers, gas adsorption and permeation, molecular sieving, lattice flexibility

## Abstract

The fabrication of molecular gas sieving materials with specific affinities for a single gas species and able to store large quantities of materials at a low or atmospheric pressure is desperately required to reduce the adverse effects of coal and oil usage in carbon capture. Fundamental understanding of the dynamic adsorption of gas, the diffusion mechanisms across thin film membranes, and the impact of interfaces play a vital role in developing these materials. In this work, single gas permeation tests across micro-porous membrane materials, based on metal organic framework crystals grown on the surface of carbon nanotubes (ZiF-8@CNT), were performed for the first time in-situ at the Australian Synchrotron on the small angle X-ray scattering beamline in order to reveal molecular sieving mechanisms and gas adsorption within the material. The results show that specific chemi-sorption of CO_2_ across the ZiF-8 crystal lattices affected the morphology and unit cell parameters, while the sieving of other noble or noble like gases across the ZiF-8@CNT membranes was found to largely follow Knudsen diffusion. This work demonstrates for the first time a novel and effective technique to assess molecular diffusion at the nano-scale across sub-nano-porous materials by probing molecular flexibility across crystal lattice and single cell units.

## 1. Introduction

Diffusion mechanisms across micro- and nano-porous structures have been demonstrated to exhibit non-conventional behaviours related to the confinement, sieving, or surface diffusion of gas or liquid molecules at the nanoscale across the pores of various materials [1,2]. New molecular gas sieving or adsorption materials with specific affinities for single gas species or able to store large volumes of gases per unit mass at low pressure are desperately required to reduce the adverse effects of emissions related to coal and oil usage [3]. The opportunities offered by ultra-high flow and ultra-selective membrane materials have motivated research towards investigating the interactions between gas and membrane pore walls within confined volumes by tuning surface molecular interactions and chemistries.

Some of the most promising micro- or nano-porous materials, whose pores act as individual channels for specific diffusion, include aligned carbon nanotubes, ordered metal oxide monoliths, and metal organic frameworks (MOFs) [4]. These high surface-to-volume ratio materials were shown to lead to enhanced flow patterns, which were attributed to the unique semi-ordered conformation of gas or liquid molecules during diffusion across the confined dimensions of the material pores [5,6]. The mechanisms of the interactions of gases and MOF crystal molecular surfaces are still poorly understood and largely evaluated on the sole basis of the theoretical architecture or experimentally evaluated morphology of MOFs [7]. However, chemisorption and macro-molecular interactions occur intrinsically across MOF materials upon gas uptake and diffusion, significantly impacting the material pore structures, surface properties, and ageing [8].

The dynamic nature of the gas uptake is affecting permeation and selectivity and the lack of understanding interactions at the sub-nanoscale is thus limiting the design of efficient MOF separation materials. Monitoring diffusion process in situ is challenging due to the fast kinetics of gas diffusion. Unfortunately, the very early events occurring within seconds cannot be followed by classical X-ray diffraction. Advanced characterization techniques to reveal dynamic adsorption of gas and diffusion mechanisms across thin film membranes are required to study the impact of interfaces for diffusion mechanisms.

It is this initial phase that now becomes accessible by combining in-situ small angle X-ray scattering (SAXS) experiments with a sub-second time resolution. Recently, the influence of solvent adsorption on materials crystallinity and swelling was also revealed by SAXS [9,10], demonstrating the uptake of organic solvent in liquid phase across ceramic. However, the in-situ determination of diffusion or adsorption kinetics across porous materials exhibiting chemical affinity for the gas molecules has not been reported to date.

**Figure A1:**
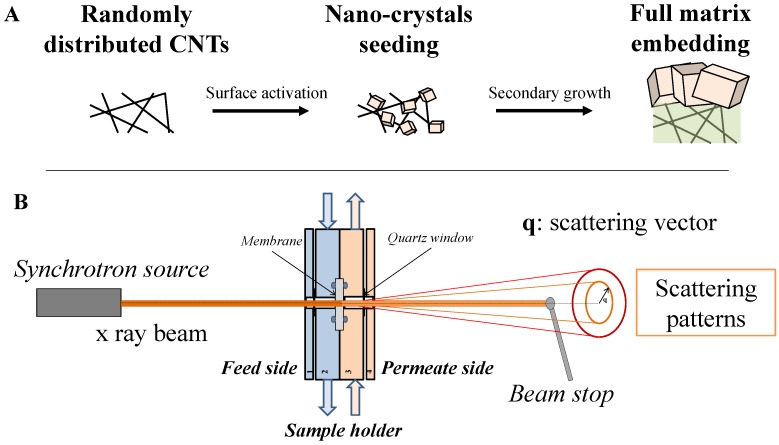
(**A**) Schematic of the MOF-CNT membranes fabrication and structure including (i) formation of a CNT BP by filtration of suspended CNTs; (ii) surface adsorption of Zn^2+^ for activation and (iii) full growth ZiF-8 crystals between the CNTs and across the BP thickness by the secondary growth method and (**B**) schematic of the SAXS acquisition process across the gas cross flow permeation module and corresponding scattering patterns formation.

Here, ZiF-8 crystal membranes, with a well-defined and narrow pore size distribution, were prepared through a simple solvo-thermal synthesis and seeded across the surface of CNTs by combining methanol solutions of zinc salts, imidazolate rings, and functionalized CNT bucky-papers (BPs) (ZiF-8@BP) [11] at room temperature by following our previous work [10]. In this paper, a novel technique to reveal interactions between nano-scale textured and porous surfaces and gas molecules, by in-situ gas permeation testing, was performed for the first time on a SAXS beamline with a Synchrotron source on defect-free ZiF-8@BP membrane composites (Figure A1). Details on the synthesis route, characterization details, and operation procedures for the in-situ SAXS operation are provided in the Appendix A.

## 2. Results and Discussion

**Figure 1:**
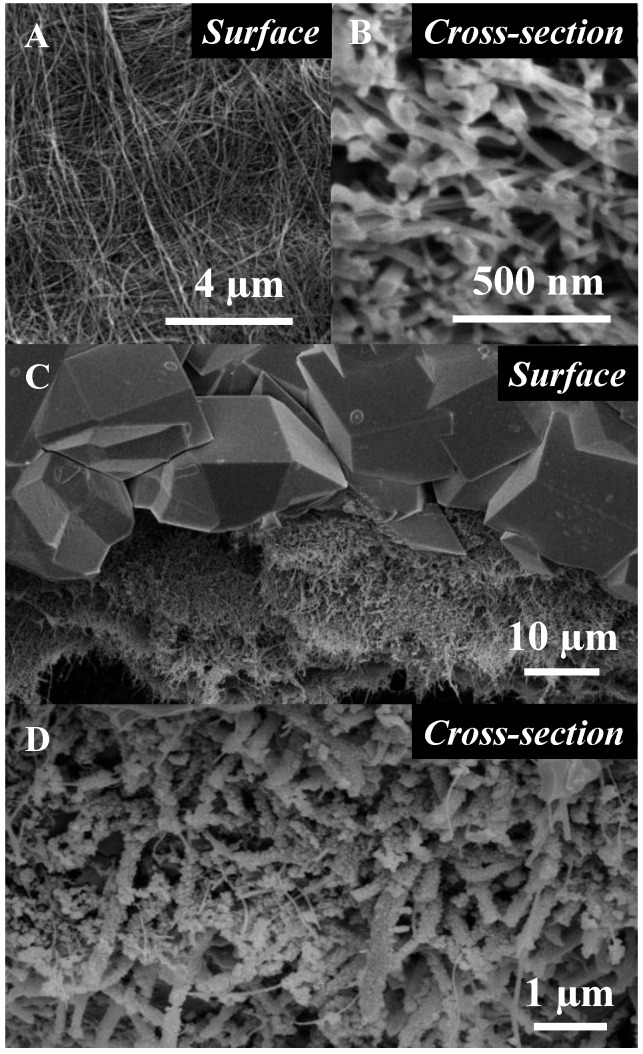
Representative scanning electron micrographs (SEMs) of the ZiF8@CNT membranes with (**A**) surface and (**B**) cross-section of a pristine CNT BP; (**C**) and (**D**) structure of the MOF membrane upon ZiF-8 growth with macron sized, acting as pore matrix for sieving, and nano-sized crystals, used for anchoring the larger ones, across the surface and cross section, respectively.

As seen in Figure 1A, the surface of the hybrid membrane materials on which we previously reported [10] was made of micron-sized crystals, partly intricate across the CNT BP mat. The carboxylic and hydroxyl groups present on the surface of the plasma functionalized CNTs [12] acted as anchoring points for the seeding and growth of nano-scale ZiF-8 nano-crystals, with diameters within a 50–100 nm range to ensure that a dense and near-defect-free matrix suitable for gas separation was formed (Figure 1B). The granular nature of the seeds allowed for the formation of strong interfaces with the surface crystals during secondary growth, which was beneficial to the handling and long-term stability of MOF materials, intrinsically particular and brittle in nature.

**Figure 2:**
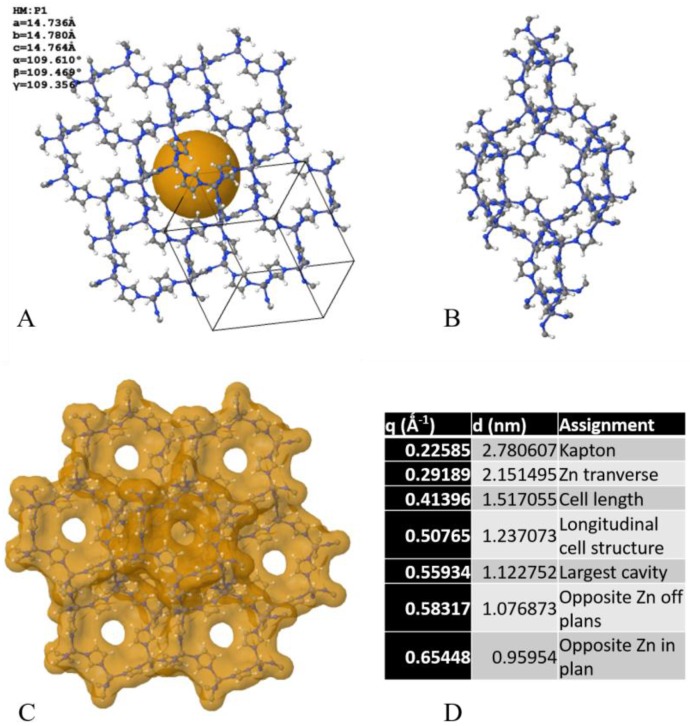
(**A**) Front view of the crystallographic structure of ZiF-8 and (**B**) side view of the cell, corresponding to a cubic body-centred structure; (**C**) corresponding pore structure and molecular surface for adsorption [17], with (**D**) equivalent scattering vectors peak positions assignments and critical dimensions modelled and calculated with [18].

As seen in Table 1, all gases, permeating across the membranes followed reasonably effective Knudsen diffusion. The specific affinity and adsorption of gases may induce local swelling, matrix distortions, or molecular group swing and re-orientation, corresponding to scattering vector shifts across the crystalline structure, visible on the SAXS patterns. ZiF-8 is a sodalite-related type structure with a cubic body-centred lattice (Figure 2A,B), exhibiting a known flexible structure, with a theoretical 3.4-Å pore diameter opening onto an 11.4-Å-diameter cage [13]. Interestingly, although the permeation of Xenon that exhibited a kinetic diameter of 3.96 Å was largely reduced, a reasonable amount still permeated through, therefore suggesting a sieving radius of ZiF-8 around that value upon gas adsorption and diffusion. This effect may be attributed to a gate opening effect from the freedom of rotation of the imidazole linkers, which was called the swing effect [14]. CO_2_ permeation was shown to be hindered compared to its theoretical Knudsen diffusion factor, which may be attributed to the enhanced enthalpy of adsorption of CO_2_ on ZiF-8 compared to noble or noble-like gases [10]. This enhanced affinity for CO_2_ might be related to a first-order adsorption and coordination in the sites of both the Zn ions and amine groups present across the imidazolate compound [15].

**Figure 3:**
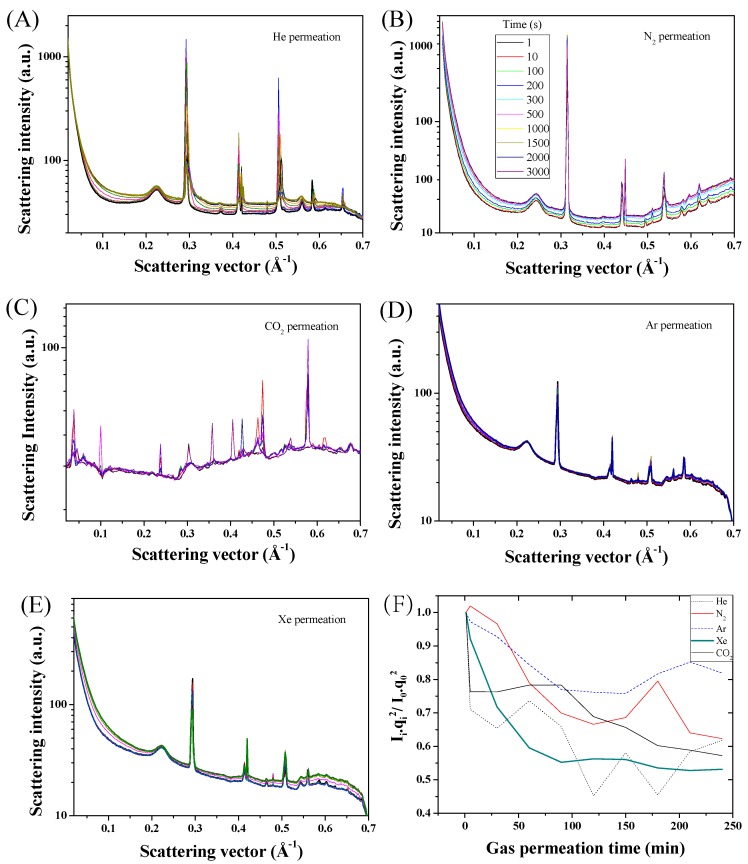
(**A**–**E**) Example of integrated scattering data shifts for the ZiF-8@BP membranes for the series of gas tested (A—He, B—N_2_, C—CO_2_, D—Ar, and E—Xe). (**F**) Correlation between the normalized I$q_{i}^{2}$/I$q_{0}^{2}$ and the permeation of the different gas for the series of membranes at q = 0.52 Å^−1^ corresponding to the (110) of the ZiF-8 lattice. Part of the intensity changes may, however, be attributed to the background change over time, due to the increase of pressure on the permeate side, and clear peak shifts occurred at various scattering vectors corresponding to the critical dimensions of the ZiF-8 crystal units. The color schemes are the same for all the shown data taken at fixed permeation times.

**Figure A2:**
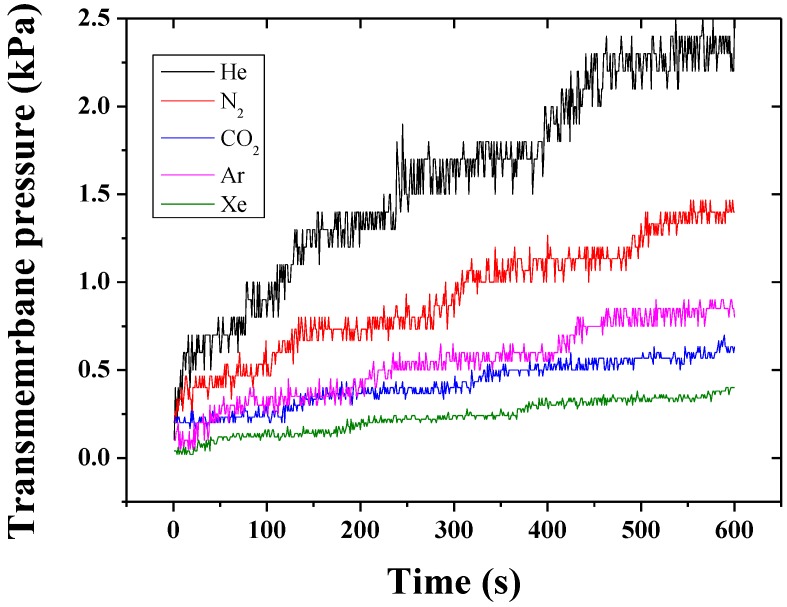
Representative permeation for short times across ZiF-8@BP membranes for the series of gases tested (He, N2, CO_2_, Ar, and Xe).

**Figure A3:**
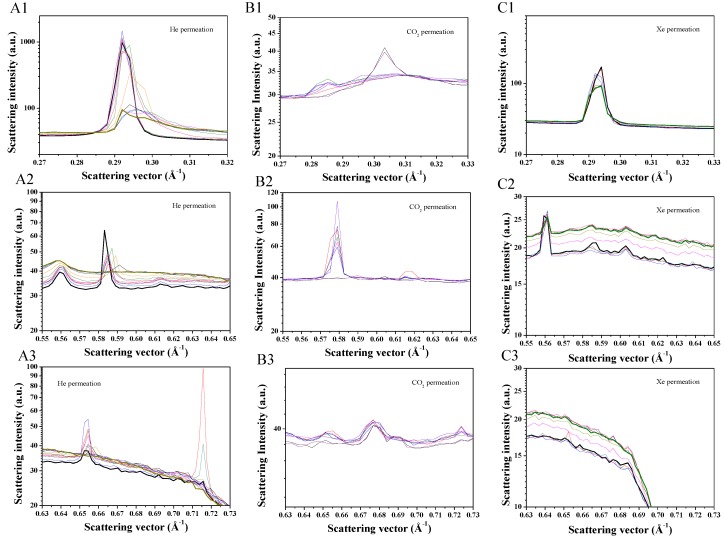
Examples of enlarged sections of the peaks discussed in Figure 3 for clarity of the shifts and intensity attenuations for the He, CO_2_, and Xe gases for the windows 0.27–0.33, 0.55–0.65, and 0.63–0.73 Å^−1^.

**Figure A4:**
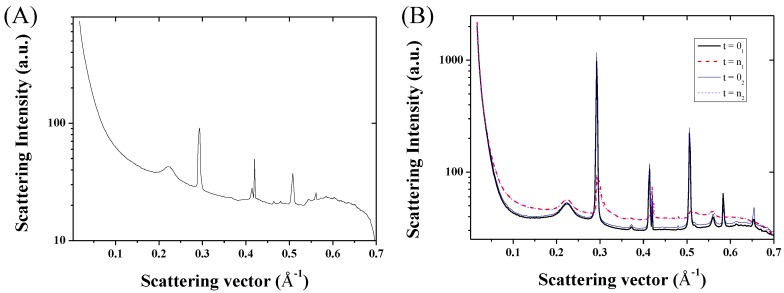
(**A**) Typical SAXS pattern for a ZiF-8@CNT membrane in air at 1 atm feed and permeate pressure; (**B**) Examples of permeation and peak recovery across the ZiF8@CNT membranes for He permeation corresponding respectively to t = 0_1_, the first duty cycle of pressurization, t = n_1_, the end of the first cycle, t = 0_2_, corresponding to the start of the second cycle, and t = n_2_, the end of the 2nd cycle.

The time-resolved reduced SAXS patterns acquired for the series of gases tested are displayed in Figure 3A–E. The time-based series corresponded to an overall increase of the transmembrane pressure of approximately 5 kPa (Figure A2), therefore relevant to the initial surface wetting of the molecular surfaces of ZiF-8 by the gases, shown in Figures 2C,D, and to a likely near-complete opening of the material pores. The adsorption enthalpy of CNTs for these gases under these low temperature and pressure conditions should lead to no major changes across the CNT [16], and no variations were visible for the Guinier knees corresponding to the CNTs. Gas adsorption and permeation across the ZiF-8@BP membranes led to shifts of Bragg peaks corresponding to the ZiF-8 microstructure and to variations of scattering intensity across the range 0.02–0.7 Å^−1^, accessible through the set SAXS camera length. Interestingly, the intensity of the peaks, attributed to unit cell parameters and reciprocal projections across the ZiF-8 lattices (Figure 3 and Figure A3) were found to sharply decrease and sometimes disappear during permeation, suggesting a deformation or a release of stress accumulated during vacuuming of the permeate chamber. The flexible nature of the ZiF-8 crystals previously demonstrated is here probed in situ during gas loading and due to the transmembrane pressure [14]. The re-arrangement of the coordinated arms across the structure may be related to electrostatic interactions and potential distributions within the matrix [15]. Interestingly, the overall intensity of the SAXS spectra was found to be also correlated to the absolute pressure across the membranes, with lower scattering intensities strongly related to gases exhibiting higher molecular weights. Peaks at 0.29, 0.59, and 0.65 Å^−1^, calculated distances between Zn–Zn nodes across the sub-unit crystals (Figure 3D), were found to shift and disappear over time during permeation but to be partly recovered upon release and re-test of the samples, suggesting an elastic deformation of the crystals (Figure A4). As seen in Figure 3F, the broad peak around 0.52–0.54 Å^−1^, corresponding to the (110) lattice of ZiF-8, was found to shift, over time and to be related to the transmembrane pressure increase. The shift towards higher scattering vector positions was found to occur independently of the type of gas, suggesting a compaction of the matrix upon pressurization of the membrane. Similarly, although no major peak position differences could be detected between the different noble gases or for N_2_, the CO_2_ permeation was found to affect the ZiF-8 microstructure much more strongly than that of the CNTs. These changes appeared to be permanent and the patterns could not be recovered after CO_2_ permeation, suggesting permanent chemi-sorption or plasticization through bond degradation across the frameworks (Figure 2A–E).

## 3. Materials and Methods

### 3.1. Materials and Membrane Synthesis

Multi-walled CNTs were grown by chemical vapour deposition [19]. A 1–5-nm-thick iron catalyst film was deposited onto a silicon substrate bearing a thin silicon dioxide layer. A mixture of helium (95%)–acetylene (5%) was used as the carbon feedstock and heated to between 650 and 750 °C. The CNTs typically have an outer diameter of ~10 (±2 nm) for a length of ~300 μm as shown from transmission electron micrographs in one of our previous studies [19]. The spacing between the vertically aligned CNTs was of approximately 100 nm corresponding to a CNT density on the wafer surface close to 1010 CNT·cm^−2^. The average CNT diameter was 10 nm (±2 nm) with a length of ~300 μm.

The MOF-CNT (ZiF-8) samples were prepared by growing MOFs on the surface of CNT assembled as Bucky-papers (BP) following a fabrication procedure previously detailed (Figure A1) [9]. In a typical MOF synthesis, a solid mixture of 1.078 g of zinc chloride (>99% Merck, Sydney, Australia), 0.972 g of 2-methyl-imidazole (>99%, Aldrich, Sydney, Australia) and 0.54 g of sodium formate (>99%, Aldrich) were dissolved in 80 mL of methanol (99.9%, Aldrich, Sydney, Australia) under sonication [20]. The BPs were placed vertically on a hydrophobic (Teflon) support to avoid undesired ZIF-8 precipitation on the surface of the support, and immersed into the previous solution for 20 min. The BP and solution were then transferred to a 200-mL Teflon autoclave and heated in an oven at 100 °C for 24 h. After cooling, the membranes were washed with methanol and dried for 24 h in an oven at 100 °C to remove residual humidity.

### 3.2. Gas Permeation Tests

In a typical in-situ dead-end gas permeation test, the membranes were sealed into a stainless steel module, prior to being put in vacuum on both up- and down-streams. After complete evacuation of the remaining adsorbed gases, SAXS pattern acquisition started immediately prior to pressurizing the up-stream line. This strategy allowed for the control of both transmembrane pressure and degassing conditions. 

Single gas permeation tests were performed with He, N_2_, CO_2_, Ar, and Xe at a 101-kPa feed to permeate transmembrane pressure difference and 25 °C in a single gas permeation rig (Figure A1) [2]. The tests were performed by placing the membrane in an O-ring sealed (25.4-mm diameter) holder, which separates a large upstream (feed—14 L) vessel from a much smaller downstream (permeate—15 mL) vessel. To ensure integrity of the O-ring seal, leak rate checks were performed with gas control impermeable poly(ethylene terephthalate) (PET) films. The area of the exposed membranes was ~5 mm^2^. All membranes were sealed onto a gas impermeable PET film with an epoxy resin and cured at room temperature for at least 12 h. After loading the membrane, both the feed and permeate vessels were evacuated under vacuum (target pressure > 0.1 kPa). The feed vessel was then isolated from both the vacuum and membrane holder, and filled to the target pressure at 101 kPa at ±0.5 kPa with analytical grade, dust-free, de-humidified gas. Prior to testing, the membrane was conditioned for 1 h under vacuum to desorb any remaining water moisture or strongly adsorbed gas. For testing, the permeate side was isolated from the vacuum. The feed vessel was opened to the permeate vessel via the membrane, and the pressure rise in the permeate vessel was monitored over time until equilibrium was reached. The feed pressure remained essentially constant due to its much larger volume compared to that of the permeate vessel. The membrane permeance was then determined from the pressure rise as a function of time 4. All the tests were performed at controlled temperature (22 ± 1 °C).

### 3.3. Characterization Techniques

Scanning electron microscopy (SEM) was performed on a Zeiss Supra 55 VP FEG Scanning Electron Microscope (Zurich, Switzerland). Micrographs were taken at a 3.5-mm working distance and under a 5-keV beam voltage (aperture 20 mm). The samples were carbon-coated with a 1–2-nm-thick layer.

SAXS experiments were performed on the SAXS/WAXS beamline at the Australian Synchrotron under the grant M5826 in March 2013. The samples were mounted within a specially designed stainless steel gas tight module with two parallel quartz windows reinforced with Kapton tape, as presented in Figure A2, and fitted onto the previously described single gas permeation rig. The high brilliance and coherence of the beam allowed for a very short acquisition time (300 ms). The energy of the beam was set at 11 keV, and scattering from the Kapton tape was determined independently and removed as background from the signals. The camera length of this series of test was 0.6 m, and the wavelength of the collimated beam was 1.0332 Å beam energy of 9.8 keV. The size of the X-ray beam was 150 μm × 250 μm. The small scattering angle, q, was inversely proportional to the scatterer diameter at small scattering angles as per Equation (1):

d ~ 0.2·π·q^−1^ (1)

where d is the space dimension of the scatterers (nm), and q is the absolute value of the scattering vector (Å^−1^).

## 4. Conclusions

This paper demonstrates for the first time insights in the specific single gas permeation experiments, correlated to scattering data to evaluate both the physi- and chemi-sorption of gas molecules. This novel approach opens the door to the design of the visualization of molecular diffusion at the nanoscale with SAXS analysis and to the probing of the flexibility and compressibility of porous micro-structures single unit crystals to gas permeation behaviours. The concept and method proposed here could also be applied to the assessment of the properties of MOF crystals and grain boundaries beyond the sole scope of separation, for sensing and storage, by providing insights on the materials thermal, mechanical, and chemical stability under specific stress.

## Figures and Tables

**Table 1:** Permeation coefficients of the membranes and main properties of the gases used. The kinetic diameters, molecular weight (Mw), and theoretical Knudsen diffusion were taken from [12], while the selectivities (theoretical, αTH and experimental αXP) were calculated from the data shown in Figure A2.

Gas	Mw (g·mol^−1^)	α_TH_ over N_2_	Kinetic Diameter (Å)	Permeance (KPa·s^−1^)	α_XP_
He	2	0.26	2.6	0.0029	0.86
N_2_	28	1	3.6	0.0025	1
CO_2_	44	1.25	3.3	0.0018	1.38
Ar	39.9	1.19	3.4	0.0022	1.13
Xe	131	2.16	3.96	0.0012	2.08

## References

[1] Hinds B.J., Chopra N., Rantell T., Andrews R., Gavalas V., Bachas L.G. (2004). Aligned multiwalled carbon nanotube membranes. Science.

[2] Sears K., Dumée L., Schütz J., She M., Huynh C., Hawkins S., Duke M., Gray S. (2010). Recent developments in carbon nanotube membranes for water purification and gas separation. Materials.

[3] Boot-Handford M.E., Abanades J.C., Anthony E.J., Blunt M.J., Brandani S., Mac Dowell N., Fernandez J.R., Ferrari M.-C., Gross R., Hallett J.P. (2014). Carbon capture and storage update. Energy Environ. Sci..

[4] Dumée L., Sears K., Mudie S., Kirby N., Skourtis C., McDonnell J., Lucas S., Schütz J., Finn N., Huynh C. (2013). Characterization of carbon nanotube webs and yarns with small angle X-ray scattering: Revealing the yarn twist and inter-nanotube interactions and alignment. Carbon.

[5] Drobek M., Bechelany M., Vallicari C., Abou Chaaya A., Charmette C., Salvador-Levehang C., Miele P., Julbe A. (2015). An innovative approach for the preparation of confined ZiF-8 membranes by conversion of ZnO ald layers. J. Membr. Sci..

[6] Drobek M., Kim J.-H., Bechelany M., Vallicari C., Julbe A., Kim S.S. (2016). Mof-based membrane encapsulated ZnO nanowires for enhanced gas sensor selectivity. ACS Appl. Mater. Interfaces.

[7] Shamsaei E., Low Z.X., Lin X., Mayahi A., Liu H., Zhang X., Zhe L.J., Wang H. (2015). Rapid synthesis of ultrathin, defect-free ZiF-8 membranes via chemical vapour modification of a polymeric support. Chem. Commun. Camb. Engl..

[8] Lau C.H., Nguyen P.T., Hill M.R., Thornton A.W., Konstas K., Doherty C.M., Mulder R.J., Bourgeois L., Liu A.C.Y., Sprouster D.J. (2014). Ending aging in super glassy polymer membranes. Angew. Chem. Int. Ed..

[9] Dumée L.F., Sears K., Schütz J.A., Finn N., Duke M., Mudie S., Kirby N., Gray S. (2013). Small angle X-ray scattering study of carbon nanotube forests densified into long range patterns by controlled solvent evaporation. J. Colloid Interface Sci..

[10] Favvas E., Stefanopoulos K., Vairis A., Nolan J., Joensen K., Mitropoulos A. (2013). In situ SAXS investigation of dibromomethane adsorption in ordered mesoporous silica. Adsorption.

[11] Dumée L., Lee J., Sears K., Tardy B., Duke M., Gray S. (2013). Fabrication of thin film composite poly(amide)-carbon-nanotube supported membranes for enhanced performance in osmotically driven desalination systems. J. Membr. Sci..

[12] Dumee L., He L., Hill M., Zhu B., Duke M., Schutz J., She F., Wang H., Gray S., Hodgson P. (2013). Seeded growth of ZiF-8 on the surface of carbon nanotubes towards self-supporting gas separation membranes. J. Mater. Chem. A.

[13] First E.L., Floudas C.A. (2013). Mofomics: Computational pore characterization of metal–organic frameworks. Microporous Mesoporous Mater..

[14] Fairen-Jimenez D., Moggach S.A., Wharmby M.T., Wright P.A., Parsons S., Duren T. (2011). Opening the gate: Framework flexibility in ZiF-8 explored by experiments and simulations. J. Am. Chem. Soc..

[15] McEwen J., Hayman J.-D., Ozgur Yazaydin A. (2013). A comparative study of CO_2_, CH_4_ and N_2_ adsorption in ZiF-8, Zeolite-13X and BPL activated carbon. Chem. Phys..

[16] Dumee L., Hill M.R., Duke M., Velleman L., Sears K., Schutz J., Finn N., Gray S. (2012). Activation of gold decorated carbon nanotube hybrids for targeted gas adsorption and enhanced catalytic oxidation. J. Mater. Chem..

[17] Novaković S.B., Bogdanović G.A., Heering C., Makhloufi G., Francuski D., Janiak C. (2015). Charge-density distribution and electrostatic flexibility of ZiF-8 based on high-resolution X-ray diffraction data and periodic calculations. Inorg. Chem..

[18] Greeves N. ZiF-8 Metal Organic Framework. http://www.chemtube3d.com/solidstate/MOF-ZIF8.htm.

[19] Huynh C.P., Hawkins S.C. (2010). Understanding the synthesis of directly spinnable carbon nanotube forests. Carbon.

[20] Merenda A., des Ligneris E., Sears K., Chaffraix T., Magniez K., Cornu D., Schütz J.A., Dumée L.F. (2016). Assessing the temporal stability of surface functional groups introduced by plasma treatments on the outer shells of carbon nanotubes. Sci. Rep..

